# Isolated Tracheal Oesophageal Congenital Fistula: A Case Report

**DOI:** 10.7759/cureus.38758

**Published:** 2023-05-09

**Authors:** Hicham Azam, Abdelouhab Ammor, Houssain Benhaddou

**Affiliations:** 1 Department of Pediatric Surgery, Mohammed VI University Hospital, Oujda, MAR

**Keywords:** trachea, surgery, malformation, fistula, esophagus

## Abstract

Esotracheal fistula is a rare malformation represented by a thin ascending channel between the esophagus and the posterior surface of the trachea. Diagnosis is sometimes difficult due to the atypical character of the symptomatology. Diagnosis is made by a gastro-duodenal oesophageal transit (TOGD) and the treatment is surgical.

We report a case of isolated congenital esotracheal fistula collected in the pediatric visceral and urogenital surgery department at the Mohammed VI University Hospital Center in Oujda, Morocco, previously not discovered, and its surgical treatment as well as an updated literature review of this entity.

## Introduction

Isolated esotracheal fistula (ETF), without esophageal atresia, called H or N fistula is a rare congenital malformation, it represents approximately four percent of all esotracheal malformations. Early diagnosis of this condition is crucial for a better prognosis, even though it can be challenging to diagnose at times, and the treatment is surgical [1,2]. We present our experience with a case of isolated congenital ETF in the pediatric visceral and urogenital surgery department at the Mohammed VI University Hospital Center in Oujda, Morocco.

## Case presentation

**Figure 1:**
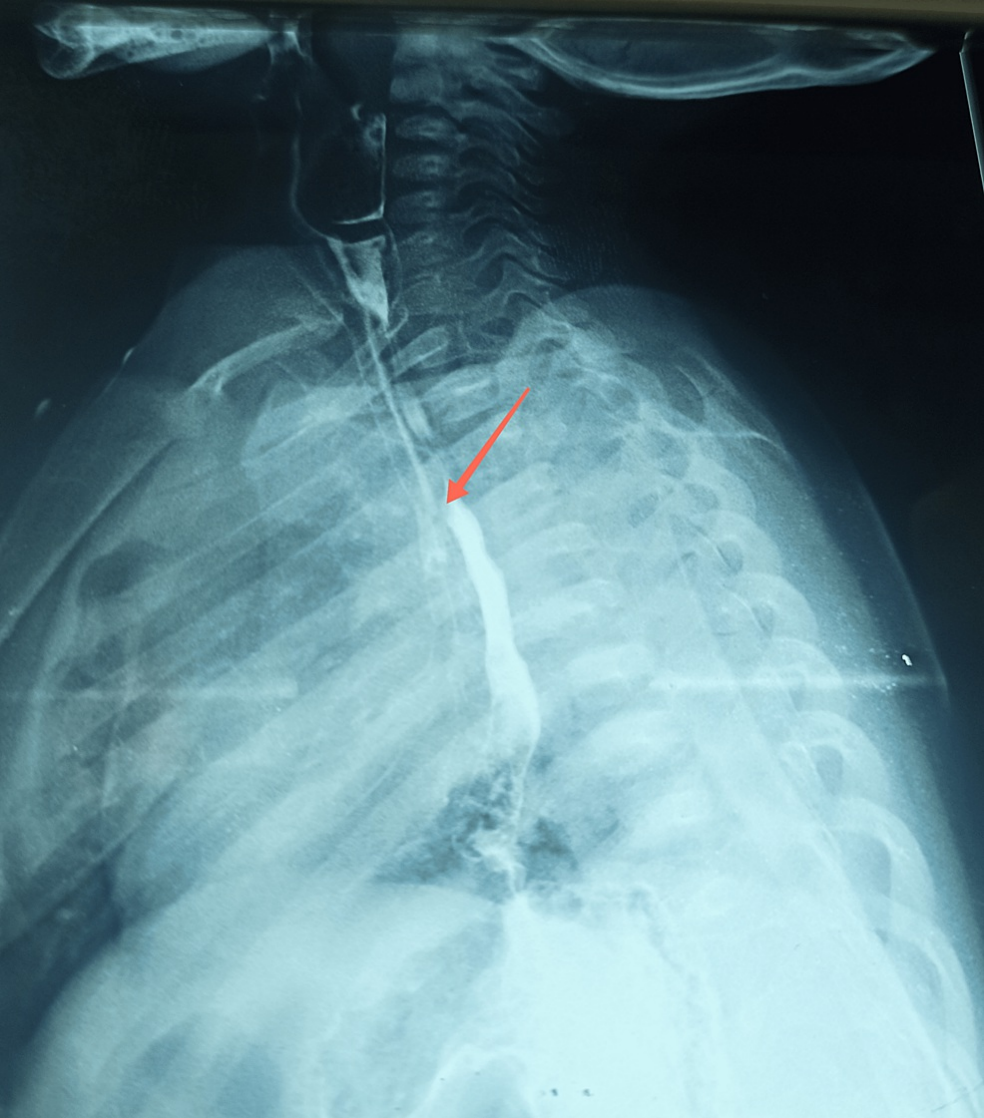
Esophagogastroduodenal transit (TOGD) Eosotracheal fistula

This was a nine-month-old infant, who has received all recommended vaccinations to date. The history of the disease dates back to birth with recurrent choking episodes and bronchoalveolitis treated repeatedly with pain relief medication, antibiotics, and antitussives without improvement. The child was referred to pediatrics for suspected gastroesophageal reflux. Radiological studies are front chest X-ray with an esophagogastroduodenal transit (TOGD) was performed showing an isolated ETF (Figure 1).

**Figure 2:**
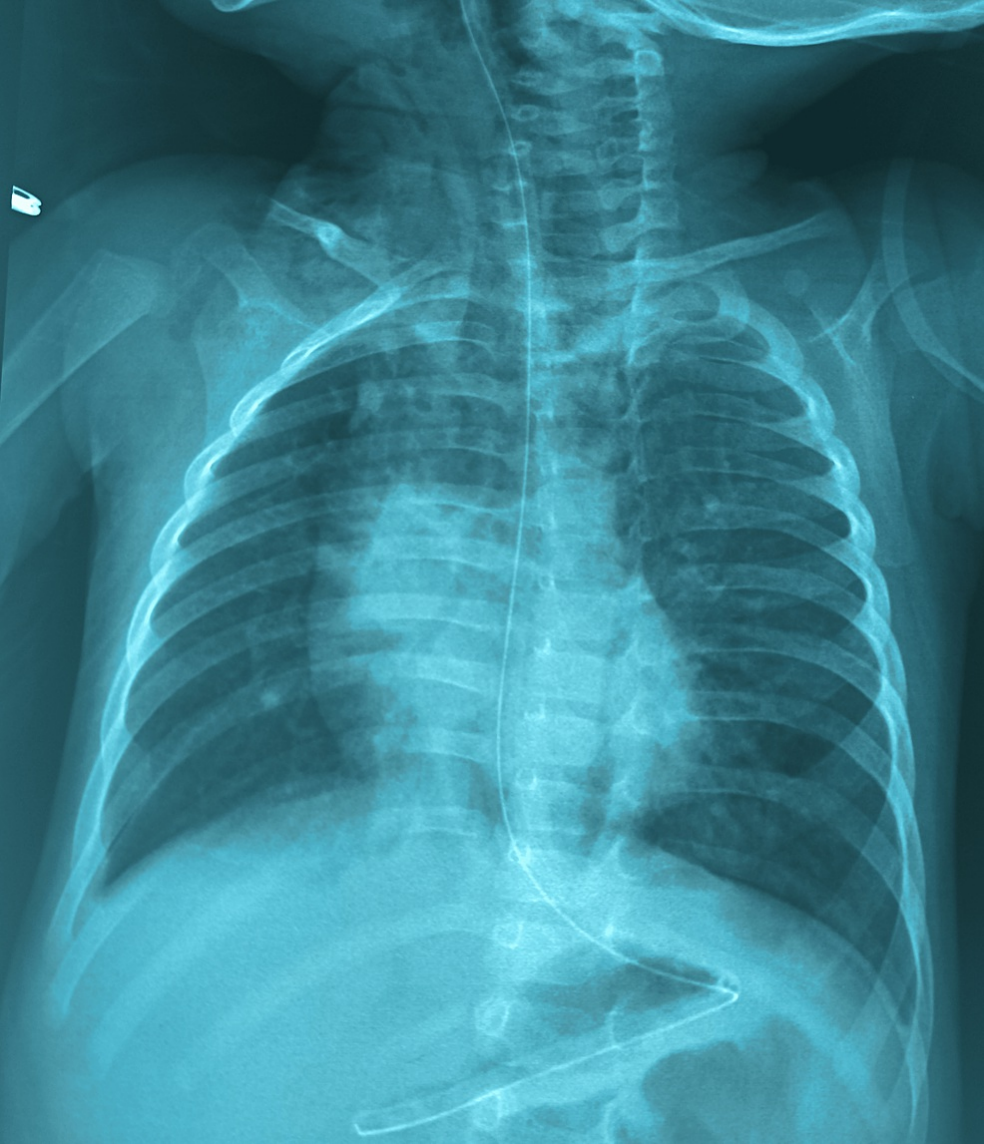
Chest X-ray Normal

Upon admission, the infant was conscious and tonic, with delayed growth and slight abdominal distension. Respiratory system examination, snoring sounds, and bilateral rales were noted, and the chest X-ray was unremarkable (Figure 2).

**Figure 3:**
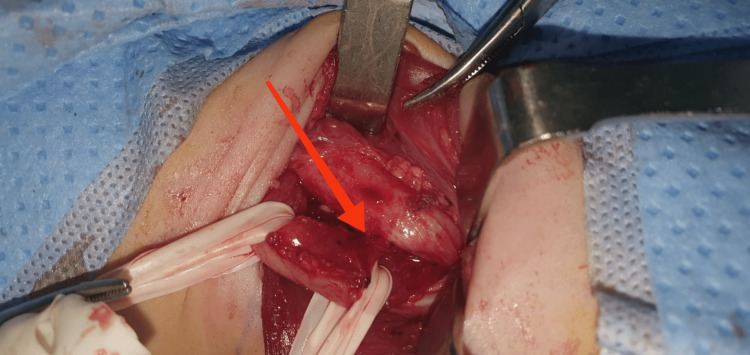
Intraoperative aspect of the fistula Esotracheal fistula, cervical location

The infant underwent a preoperative bronchoscopy, revealing the fistula and its cervical location. A right transverse cervical incision was made, allowing for the resection of the fistula, suture of the esophagus tear, and trachea with muscular interposition (Figure 3).

**Figure 4:**
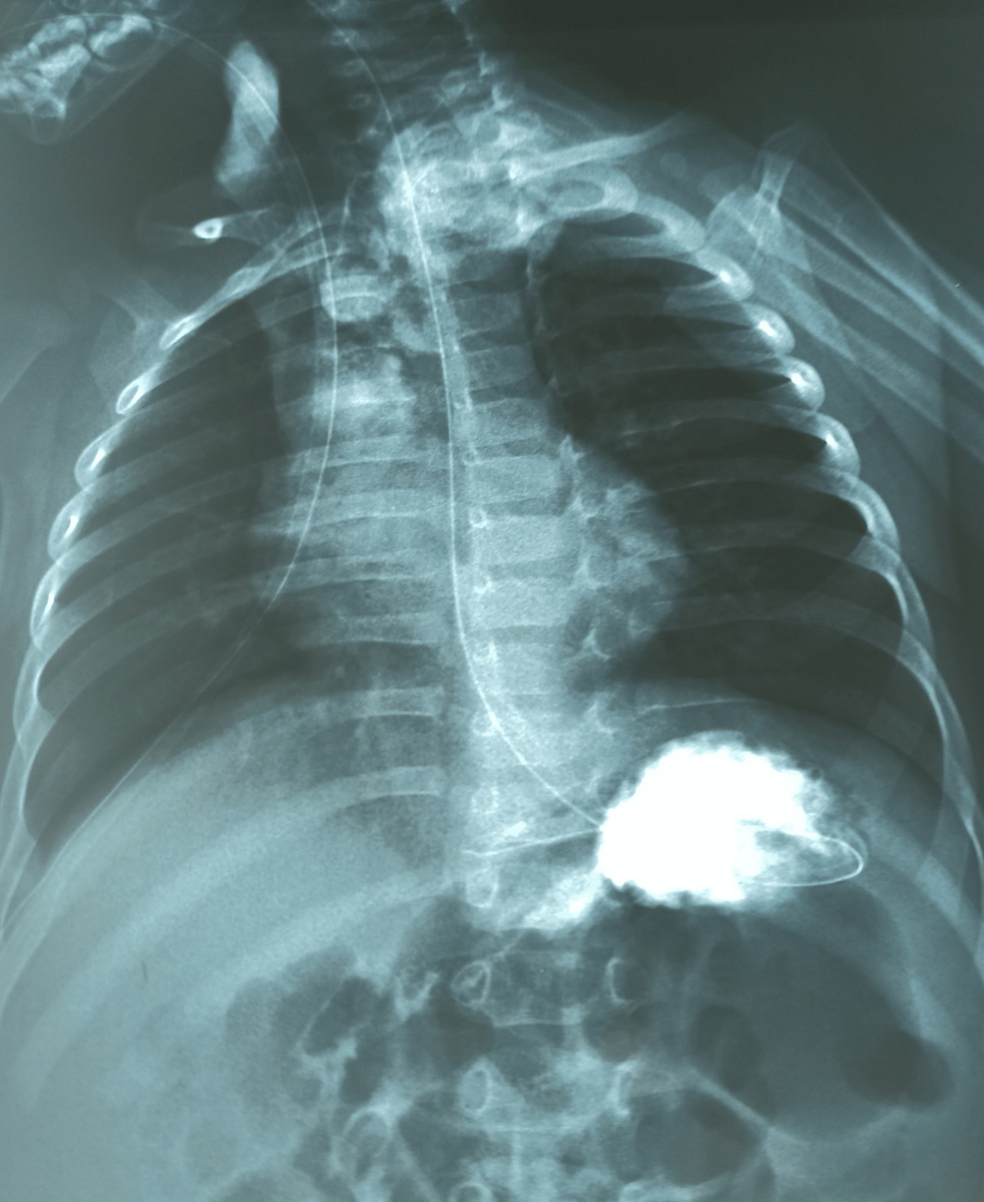
Absence of fistula on TOGD on D10 postoperative TOGD: esophagogastroduodenal transit

The postoperative course was marked by the onset of respiratory distress on day 10 after oral feeding was started (Figure 4).

**Figure 5:**
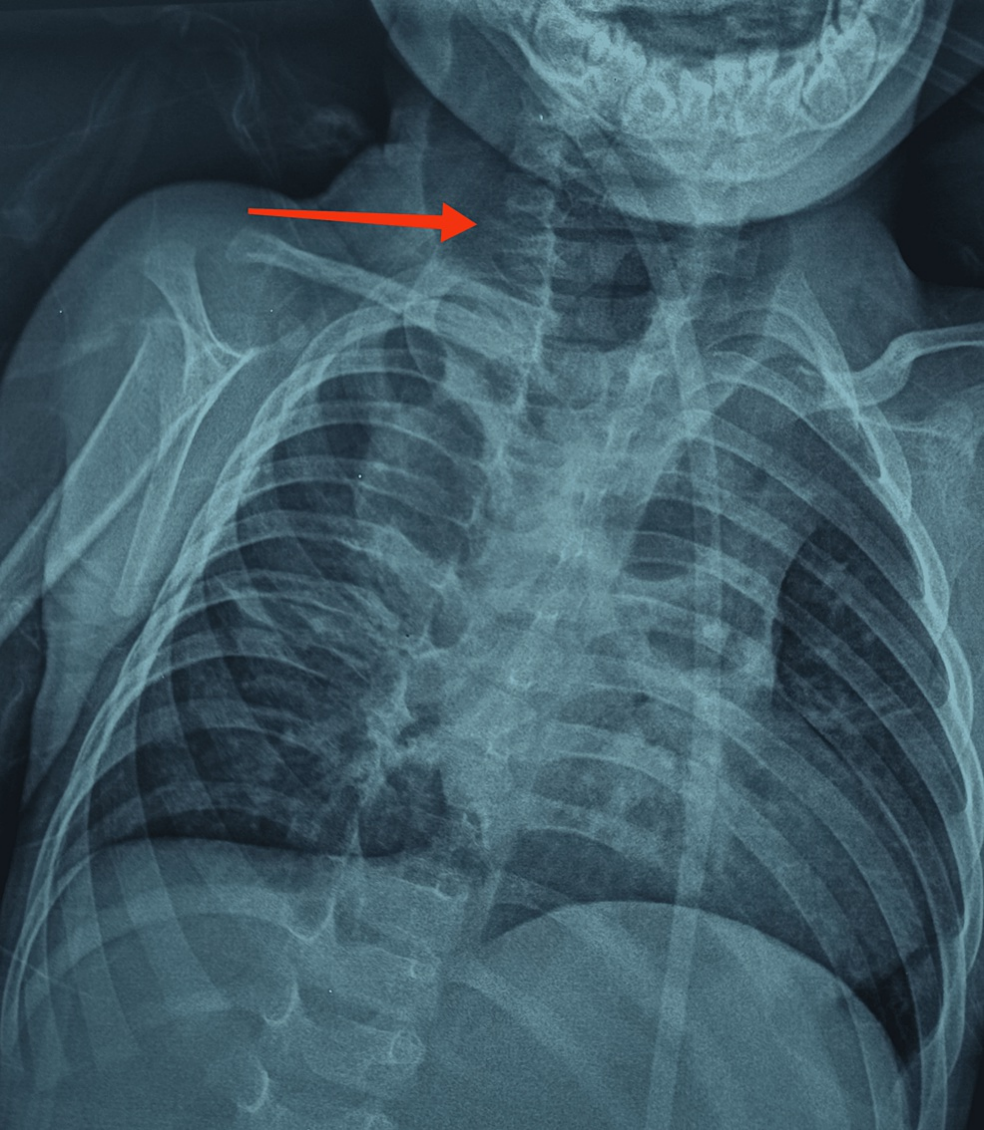
Cervical emphysema bubble on chest X-ray

**Figure 6:**
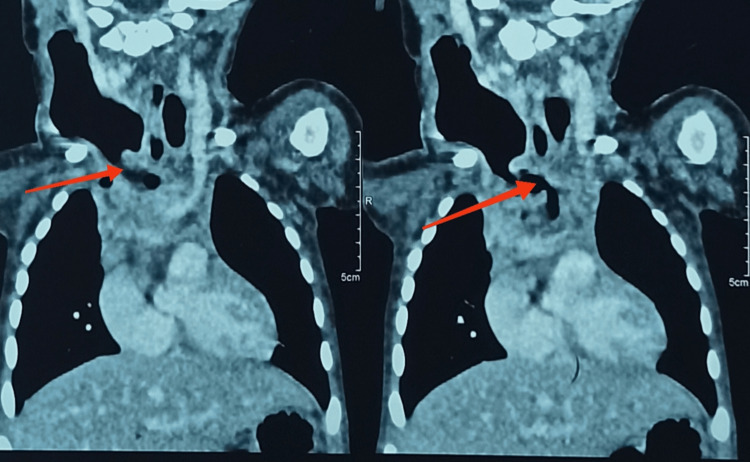
Defect in the right lateral wall of the esophagus

Clinical examination revealed a reddish cervical swelling with crepitation at the site of the cervical incision scar. Radiological testing showed a cervical emphysema bubble with a defect in the right lateral wall of the esophagus at the level of the first thoracic vertebra (D1), supplying a cervical-mediastinal abscess collection, and no identification of an ETF tract (Figure 5 and Figure 6).

Fistula repermeation was observed, leading to the decision to redo the surgery. Intraoperative exploration revealed a purulent collection in contact with the esophagus with an occluded esophageal and tracheal fistula. Closure of the esophageal defect, drainage, and gastrostomy for feeding were performed.

**Figure 7:**
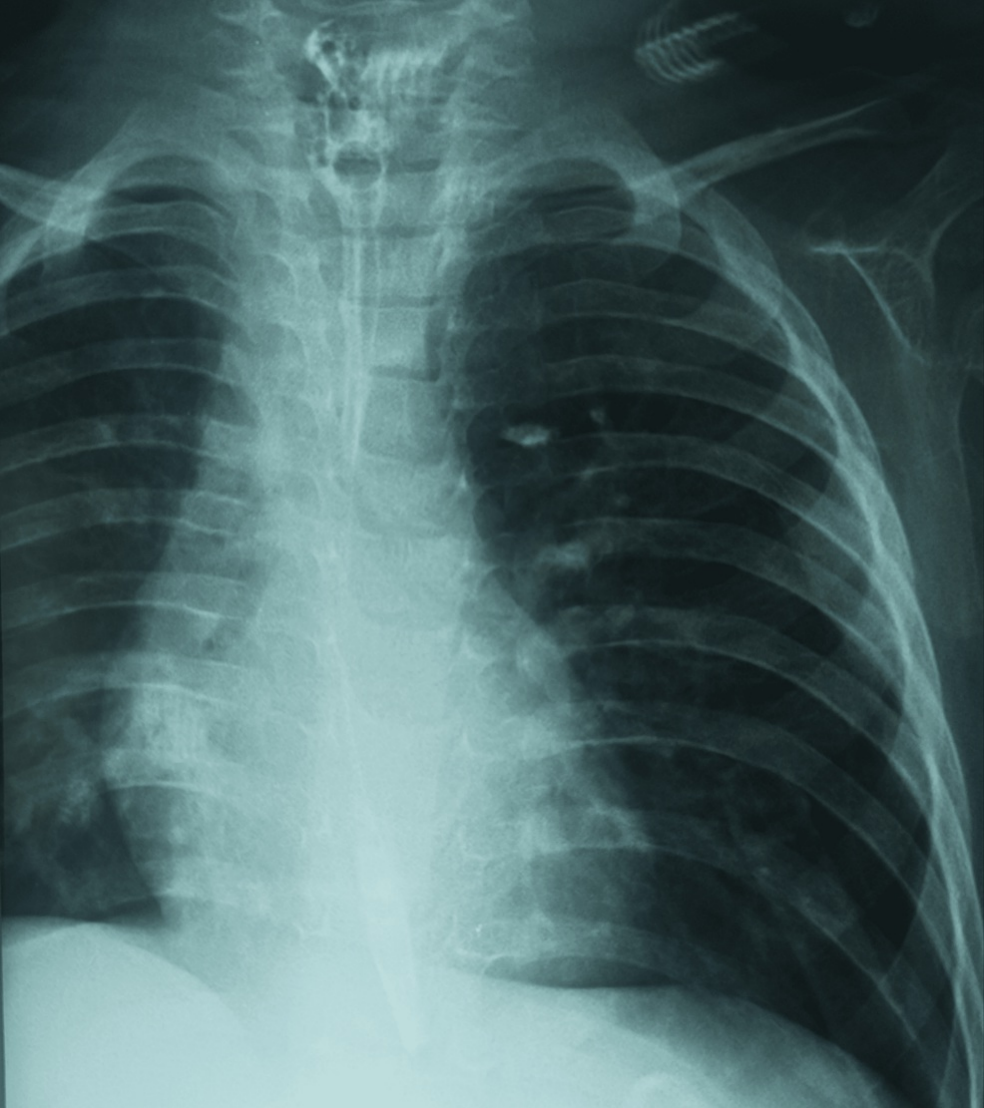
Absence of fistula on TOGD two months postoperative TOGD: esophagogastroduodenal transit

The second post-op course was uneventful, with feeding via gastrostomy without incident. At two months postoperatively, the follow-up TOGD showed no fistula, and oral feeding was started (Figure 7).

At the four-month postoperative follow-up, the infant was found to be normal, with appropriate weight, no respiratory signs, and the gastrostomy spontaneously closed.

## Discussion

The H-type fistula embryologically represents the least severe form of laryngotracheoesophageal clefts. Its incidence is estimated to be between one in 50,000 and one in 80,000 live births. It is the result of incomplete development of the tracheoesophageal septum between the fourth and eighth week of gestation. Genetic, environmental, and teratogenic factors are believed to play a role [1-4].

Associated malformations are common and similar to those observed with esophageal atresia, and sometimes are part of the VACTERL syndrome. The site of the fistula is usually cervical, and it connects the esophagus and trachea in an ascending pathway from back to front, with a short caliber of a few millimeters [1,2,5].

The pathognomonic clinical signs consist of a triad that includes coughing during feeding, abdominal distention, and recurrent pneumonia. This triad is rarely completely described. Only coughing and choking while drinking are consistently observed. The diagnosis is made in 80% of cases before the age of one year, but it can be challenging and delayed. The differential diagnosis is made with other causes of recurrent lung infection, such as gastroesophageal reflux, cystic fibrosis, bronchial dilation, intra-bronchial foreign body, and laryngeal cleft [1,2,4,6].

The definitive diagnosis relies on direct visualization of the fistula by contrast radiography. A kinetic study by cineradiography is currently the most recommended [1,3,4]. Repetition of this exam is crucial. Beasly and Meyers, reporting on a series of 30 H-type fistulas, found that the diagnosis was made in 73% of cases with the first contrast study, and in 100% of cases with the third attempt for some patients [7].

Currently, the complementary technique that is unanimous is tracheoscopy, which some authors couple with esophagoscopy. During this exam, methylene blue can be injected into the esophagus to see if it appears in the trachea. Finally, endoscopy allows for catheterization of the fistula, which will guide the surgical procedure [1-4,6,8].

The treatment of H-type fistulas is surgical. The cervical approach is always preferable for fistulas located above D2. The thoracic approach is reserved for those close to the carina [1,2].

Preoperative catheterization of the fistula under tracheoscopy makes its identification easy and allows for minimal dissection, some authors propose muscle-aponeurotic interpositions between the esophagus and trachea, such as pleural tissue, sternocleidomastoid muscle, thyroid muscle, thymic tissue, rectus abdominis muscle, and pericardium [2-4,6,9-12].

Postoperative morbidity can be significant, including usually transient recurrent laryngeal nerve palsy, tracheomalacia, atelectasis, fistula recurrence, and, most notably, digestive problems such as gastroesophageal reflux, motility disorders, and esophageal stenosis [1,2,4,7].

Fistula recurrence is a rare but serious complication. It is more likely a missed fistula during the initial surgery. Catheterization of the fistula by tracheoscopy and muscle interposition plasty can reduce this risk [2,9,10,12].

## Conclusions

Esophageal tracheal fistula is a rare malformation. The diagnosis should be considered in the presence of respiratory symptoms occurring during feeding, particularly in the neonatal period. If clinical evidence suggests this abnormality, complementary investigations should confirm it and may need to be repeated. Bronchoscopy can provide initial assistance with the diagnosis and prepare for surgical intervention.
